# Targeting the Immunogenetic Diseases with the Appropriate HLA Molecular Typing: Critical Appraisal on 2666 Patients Typed in One Single Centre

**DOI:** 10.1155/2013/904247

**Published:** 2013-01-21

**Authors:** M. Guarene, C. Capittini, A. De Silvestri, A. Pasi, C. Badulli, I. Sbarsi, A. L. Cremaschi, F. Garlaschelli, C. Pizzochero, M. C. Monti, C. Montecucco, G. R. Corazza, D. Larizza, P. E. Bianchi, L. Salvaneschi, M. Martinetti

**Affiliations:** ^1^Laboratorio di Immunogenetica, Servizio di Immunoematologia e Medicina Trasfusionale, Fondazione IRCCS Policlinico San Matteo, 27100 Pavia, Italy; ^2^Unità di Biometria, Direzione Scientifica, Fondazione IRCCS Policlinico San Matteo, 27100 Pavia, Italy; ^3^Clinica Reumatologica, Fondazione IRCCS Policlinico San Matteo, Università degli Studi di Pavia, 27100 Pavia, Italy; ^4^Clinica Medica I, Centro per lo Studio e la Cura delle Malattie Infiammatorie Croniche Intestinali, Fondazione IRCCS Policlinico San Matteo, Università degli Studi di Pavia, 27100 Pavia, Italy; ^5^Clinica Pediatrica, Fondazione IRCCS Policlinico San Matteo, Università degli Studi di Pavia, 27100 Pavia, Italy; ^6^Clinica Oculistica, Fondazione IRCCS Policlinico San Matteo, Università degli Studi di Pavia, 27100 Pavia, Italy; ^7^Servizio di Immunoematologia e Medicina Trasfusionale, Fondazione IRCCS Policlinico San Matteo, Università degli Studi di Pavia, 27100 Pavia, Italy

## Abstract

We compared the immunogenetic data from 2666 patients affected by HLA-related autoimmune diseases with those from 4389 ethnically matched controls (3157 cord blood donors CBD, 1232 adult bone marrow donors BMD), to verify the appropriateness of HLA typing requests received in the past decade. The frequency of HLA-B∗27 phenotype was 10.50% in 724 ankylosing spondylitis, 16.80% in 125 uveitis (3.41% BMD, 4.24% CBD, *P* < 0.0001); HLA-B∗51 allele was 15.57% in 212 Behçet's disease (12.91% BMD, 9.88% CBD, *P* < 0.0001); the HLA-DRB1-rheumatoid arthritis (RA) shared epitope was 13.72% in 554 RA (10.85% BMD, 13.48% CBD, *P* = 0.016); the carriers of almost one of HLA-DQB1 susceptibility alleles were 84.91% in 795 celiac disease (CD) and 59.37% in 256 insulin-dependent diabetes mellitus (IDDM) (46.06% in 875 CBD, 42.75% in 662 BMD *P* < 0.0001). Overall, our results show that the HLA marker frequencies were higher in patients than controls, but lower than expected from the literature data (excluding CD and IDDM) and demonstrate that, in complex immunogenetic conditions, a substantial number of genetic analyses are redundant and inappropriate, burdening to the public health costs. For this reason, we suggest the Italian Scientific Society of Immunogenetics to establish guidelines to improve the appropriateness of typing requests.

## 1. Introduction

An autoimmune disease arises when the immune system looses the ability to distinguish body's own cells from foreign cells (nonself), thus eliciting the attack of self-tissues [1]. From a genetic point of view, an autoimmune condition is the result of a tight interaction between environmental factors and specific predisposing genes. The first autoimmune disease found to be associated with an HLA-B marker was ankylosing spondylitis (AS), a chronic and progressive inflammation of the spine articulations, and less frequently the peripheral joints, leading to a forward-stooped posture which causes rigidity and severe pain [2, 3]. After almost four decades, AS is still tightly associated with HLA-B*27 with a phenotype frequency of 90% [4].

From then onwards, the susceptibility to develop autoimmune diseases has been found to be conditioned by several HLA molecules (Table 1). In ocular autoimmune conditions, diseases of the uvea show the strongest correlation with HLA markers. Uveitis is an inflammation of the uvea that destroys eye tissues, causes light sensitivity, and decreases visual acuity [5, 6]. Acute anterior uveitis is described as strictly associated to HLA-B*27 (phenotypic frequency 50%) [7]. Behçet's disease (BD) is a chronic vasculitis characterized by aphthosis, uveitis, and skin lesions, and sometimes affecting also the musculoskeletal, nervous, and gastrointestinal systems [8]. In Southern Europe, the HLA-B*51 allele accounts for a 30–50% of the genetic risk for BD development and is carried by one- to two-thirds of patients all over the world (Caucasian phenotypic frequency 60–70%) [9]. Rheumatoid arthritis (RA) is a chronic and systemic inflammatory disease affecting the joints, and gradually leading to their destruction [10–12]. Since the Seventies, the RA risk has been associated to specific HLA-DRB1 molecules (HLA-DRB1*01, *04, *10) and, more recently, to few variants sharing an amino acid sequence (position 70–74) in the third hypervariable region of HLA-DR *β* chain known as Shared Epitope (SE) (Table 1) [13]. Celiac disease (CD) is a chronic enteropathy caused by gluten intake in people carrying the HLA predisposing variants coding for the DQ2 (HLA-DQB1*02:01 and HLA-DQB1*02:02) and DQ8 (HLA-DQB1*03:02) molecules [14–16]. The phenotype frequency of DQ2/8 is 20% in healthy people and more than 90% in CD patients [17]. Insulin-dependent diabetes mellitus (IDDM) is a chronic autoimmune disorder in which the immune system attacks and destroys the beta-cells of pancreas, leading to insulin dependence [18]. The HLA markers of IDDM are HLA-DQB1*02:01 (part of the DR3 haplotype: DRB1*03-DQB1*02:01) and HLA-DQB1*03:02 (part of the DR4 haplotype: DRB1*04-DQB1*03:02) [19]. About 40–60% of patients with IDDM carry the DR3/4-DQ2/8 genotypes [20]. 

Since 1989, the Immunogenetics Laboratory of the IRCCS Policlinico San Matteo of Pavia (Italy) is involved in the HLA molecular typing of hematopoietic stem cell donors and patients affected by autoimmune diseases. In particular, the HLA typing requests for autoimmune diseases come from both medical specialists (inside and outside the IRCCS Policlinico San Matteo) and general practitioners, to support their clinical diagnosis. The collaboration between the physicians and the immunogenetists is fundamental to reach the most appropriate typing request. In fact, thanks to the tight correlation between specific HLA markers and the predisposition to certain autoimmune diseases, the HLA analysis has become a sensitive genetic tool to identify those individuals at high risk to develop these conditions [21].

A recent survey, performed by the Italian Society of Immunogenetics (AIBT), has shown that the HLA analyses, requested for disease association studies, are plethoric and often inappropriate (unpublished data). Moreover, HLA typing technologies adopted in the immunogenetics laboratories throughout Italy are heterogeneous. Trying to overcome the waste of economic resources for useless investigations, in 2010 the AIBT has proposed a long-distance course addressed to Italian operators in the immunogenetics field.

Taking into account these premises, we have retrospectively analyzed the HLA typing data of 2666 patients referred to our hospital for different autoimmune pathologies, and addressed to our Immunogenetics Laboratory in the last decade (2002–2011), to verify the appropriateness of the typing requests, comparing the frequency of HLA variants with those of 4389 ethnically matched controls typed in the same period with the same molecular techniques.

## 2. Materials and Methods

### 2.1. Patients

The Immunogenetics Laboratory of the IRCCS Policlinico San Matteo (Pavia) is accredited by the EFI (European Federation of Immunogenetics) since 1998 for stem cell transplantation and HLA disease association studies and daily receives HLA typing requests from physicians to support the suspected diagnosis. The well-recognized HLA susceptibility markers of ankylosing spondylitis, rheumatoid arthritis, uveitis, Behçet's disease, celiac disease, and insulin-dependent diabetes mellitus are listed in Table 1.

HLA typing requests also come from the other hospitals of Pavia and Province as well as from general practitioners. Thus, we could not verify the compliance of the classification criteria conventionally recognized for the diagnosis of the six autoimmune pathologies considered in the study (Table 1) [22–27]. In detail, Table 2 shows the percentage of HLA typing requests coming from medical specialists (inside and outside the Policlinico San Matteo of Pavia) and general practitioners.

We considered 724 HLA typing data for patients affected by ankylosing spondylitis (mean age 44.3 years, female/male rate 1.5), 554 with rheumatoid arthritis (mean age 52.2 years, female/male rate 2.8), 125 with uveitis (mean age 41.9 years, female/male rate 0.9), 212 with Behçet's disease (mean age 41.7 years, female/male rate 2.1), 795 affected by celiac disease (mean age 20.5 years, female/male rate 1.5), and 256 with type I diabetes (mean age 10.3 years, female/male rate 1.0). All these immunogenetic data were collected from 2002 to 2011. 

### 2.2. Control Subjects

Two control groups were considered: 3157 cord blood (CB) donors belonging to the Pavia CB Bank (IRCCS Foundation Policlinico San Matteo, Pavia, Italy) and 1232 adult hematopoietic stem cell donors belonging to the PV01 Registry (Italian Bone Marrow Registry, IBMDR, Genoa, Italy). All the individuals were of Caucasian ancestry. The mothers of each CB donor signed an informed consent to participate in the CB banking program for unrelated stem cell transplantation. Following the international protocols, the physicians obtained a complete medical history of the newborn's family (mother, father, siblings, and grandparents). An exhaustive obstetric history (i.e., of previous pregnancies) was also obtained, so that we could exclude women with recurrent spontaneous abortions from donation. Furthermore, during each pregnancy, the health of both mother and fetus was carefully monitored, in order to exclude from donation any CB unit derived from pathological pregnancies, including preterm ones (<37 gestational weeks). All the adult hematopoietic stem cell donors signed an informed consent to be enrolled in the Italian Bone Marrow Registry. The IBMDR criteria exclude from donation individuals affected by genetic, cardiovascular, hematologic, autoimmune and psychiatric conditions, malignancies, and infectious diseases.

### 2.3. HLA Classes I and II Genomic Typing

The entire data set of patients and controls is composed by molecular typing for both HLA class I and II genes. The PCR sequence-specific primer method (Olerup, Sweden) and/or the reverse PCR sequence specific oligonucleotide hybridization method (Innogenetics, Murex Biotech Limited, Belgium) were employed, as described elsewhere [28]. Ambiguous typing results were resolved by direct sequencing.

Patients affected by ankylosing spondylitis were typed for the presence of the HLA-B*27 allele, those affected by rheumatoid arthritis and uveitis for the HLA-B and HLA-DRB1 loci, and patients affected by Behçet's disease for the HLA-B locus. The polymorphisms at the HLA-DQA1 and HLA-DQB1 loci were defined at high resolution in individuals affected by celiac disease and type I diabetes.

According to the international policy of FACT-Netcord and Italian CB Banks, the CB units were typed for HLA-A and HLA-B polymorphisms at low-resolution level and for HLA-DRB1 at high-resolution level before banking. According to the policy of IBMDR, the adult hematopoietic stem cell donors were typed for HLA-A and HLA-B and, more recently, for HLA-C polymorphisms at low-resolution level and for HLA-DRB1 at high resolution. We analysed the HLA-B polymorphisms at low-resolution level and the HLA-DRB1 polymorphisms at high-resolution level for 3157 CB donors and 1232 adult hematopoietic stem cell donors. We analysed the HLA-DQA1 and HLA-DQB1 polymorphisms at high-resolution level for 875 CB donors and 662 adult hematopoietic stem cell donors.

### 2.4. Statistical Analysis

Differences between the groups were evaluated with Chi-squared statistics or Fisher's exact test, as appropriate. *P* values reported were two tailed; *P* < 0.05 was considered statistically significant.

We employed the Principal Coordinates Analysis (PCoA) statistical method to explore and visualize similarities or dissimilarities between patients and controls. PCoA starts with a similarity matrix (correlation) or dissimilarity matrix (distance matrix) and assigns for each population a location in a low-dimensional space, for example, as a 2D or a 3D graphics. PCoA involves projecting the points onto a space defined by a small number of principal axes (uncorrelated linear combinations of the variables that contain most of the variance), accounting for the greatest variability. The first axis accounts for the highest variance and the second axis for the lowest one. The samples are represented by points, and the proximity among them shows their similarity or dissimilarity [29].

## 3. Results and Discussion

In the past decade, our Immunogenetics Laboratory received a growing number of typing requests (500 in 2011) for patients in the setting of HLA-related autoimmune diseases. Aiming at verifying the appropriateness of all these genetic analyses in helping physicians to define the clinical diagnoses, we retrospectively analyzed the HLA typing data of 2666 autoimmune patients that we collected from 2002 to 2011, comparing the frequency of HLA markers with those of 4389 ethnically matched controls (3157 cord blood donors CBD and 1232 adult bone marrow donors BMD).

**Figure 1:**
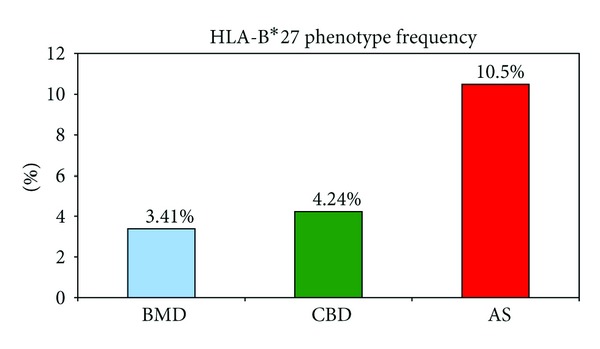
Phenotype frequency of HLA-B*27 in ankylosing spondylitis (AS) patients, adult bone marrow donors (BMD), and cord blood donors (CBD).

HLA-B*27 is the molecular marker of both ankylosing spondylitis (AS) and acute anterior uveitis (AAU) (Table 1). We received 724 HLA-B*27 typing requests to support the suspected diagnosis of AS based on clinical symptoms or for familial counseling. The literature reports that about 90% of truly diagnosed Caucasian AS patients carry the HLA-B*27 marker [24]. Comparing the phenotype frequency of HLA-B*27 between the patients and the two control groups, we found that 10.5% of suspected AS (sAS) patients were actually positive for HLA-B*27, whereas only 3.41% of BMD and 4.24% of CBD carried the HLA-B*27 marker (AS versus BMD *P* < 0.0001, AS versus CBD *P* < 0.0001; see Figure 1). 

We also considered 125 HLA-B*27 typing requests for Uveitis (U) patients, finding a higher HLA-B*27 allele frequency with respect to controls (8.40% uveitis versus 1.70% BMD and 2.15% CBD, *P* < 0.0001) (Table 3), corresponding to 16.80% uveitis versus 3.41% BMD and 4.24% CBD phenotype frequencies. According to several association studies, the phenotypic frequency of the HLA-B*27 marker in classic acute anterior uveitis patients is about 50% [7] whilst in our sample is only 16.80%. This result may be due to the heterogeneity of patients referring to our laboratory with a generic definition of uveitis, but probably affected by anterior, intermediate, posterior uveitis, and panuveitis.

We considered 212 requests of HLA-B low-resolution typing for Behçet's disease (BD) patients. The allelic frequency of HLA-B*51 was 15.57% slightly higher than in BMD (12.91%) and significantly increased with respect to CBD (9.88%, *P* < 0.0001) (Table 3). Once more, in our patients with a suspect of BD, we found an HLA-B*51 allele frequency lower than the expected, suggesting an extreme caution of our physicians in giving a fast and definite diagnosis of such a complex syndrome.

**Figure 2:**
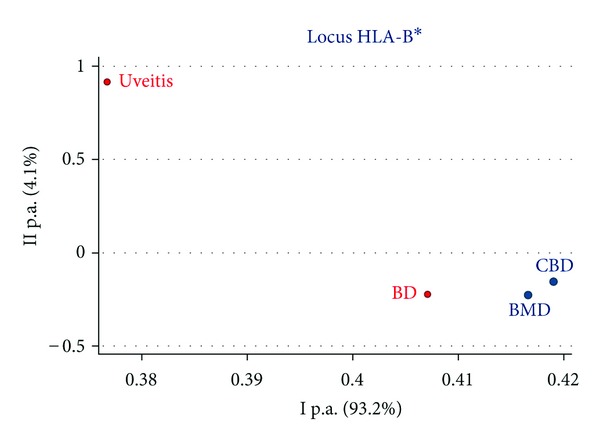
Principal component analysis of HLA-B in uveitis, Behçet's disease (BD), adult bone marrow donors (BMD), and cord blood donors (CBD).

In order to visualize in a single graphic the genetic distances among groups (patients and controls), we performed the statistical test of the Principal Coordinates Analysis (PCoA): the closer the points, the more similar the samples. According to HLA-B typing, the BD patients were nearer the healthy controls than U patients (Figure 2), and this may be explained by the type of pathology, as uveitis is restricted to a single organ (the eye), whereas BD is a multiorgan syndrome affecting the nervous and gastrointestinal systems, the joints, the eye, and the skin. Interestingly, the phenotype frequency of HLA-B*51 carriers was 57.14% in the group of patients treated in neurological clinics, 28.77% in rheumatologic clinics, and 29.03% referring to general practitioners. Thus, the BD with a neurological component seems to be more clearly HLA targeted than the other clinical subtypes.

We reviewed 554 HLA-DRB1 typing requests asked to support the suspected diagnosis of rheumatoid arthritis (RA) based on early clinical manifestations. The cumulative frequency of the shared epitope (SE) alleles was 13.72%, whereas in BMD it was 10.85% (RA versus BMD *P* = 0.016) and in CBD 13.48% (Table 3). In particular, HLA-DRB1*01:01 represented the most frequent variant in RA patients (7.45%) compared to BMD (4.91%, RA versus BMD *P* = 0.005) and CBD (6.51%). In addition, HLA-DRB1*03:01 was found to be significantly more frequent in RA patients (10.05%) than in both BMD (7.47%, RA versus BMD *P* = 0.018) and CBD (7.71%, RA versus CBD *P* = 0.019). This last result is in line with the literature data, as HLA-DRB1*03:01 allele is part of the HLA-A1,B8,DR3 ancestral haplotype (AH8.1), which is the most cited haplotype in the literature for its correlation to a plethora of autoimmune diseases.

**Figure 3:**
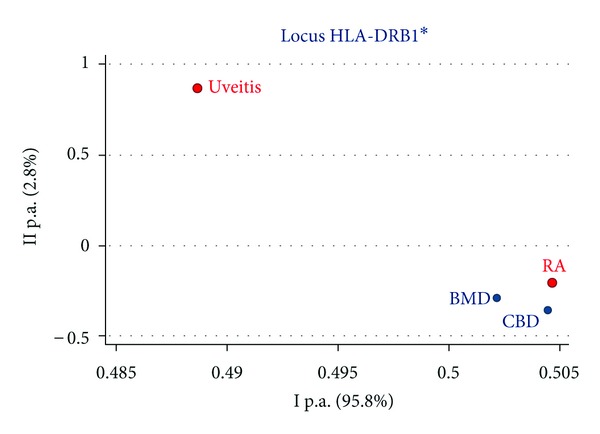
Principal component analysis of HLA-DRB1 in uveitis, rheumatoid arthritis (RA), adult bone marrow donors (BMD), and cord blood donors (CBD).

In the PCoA for the HLA-DRB1 alleles, U patients were far from both controls, while RA patients were close (Figure 3). The distance between U patients and controls highlighted the involvement of specific HLA-DRB1 markers. However, in 72 U patients we did not found a higher frequency of the well-known panuveitis markers [25], but a significant increase of HLA-DRB1*16 (12.50% U versus 7.23% BMD, *P* = 0.03; versus 6.74% CBD, *P* = 0.01). At our knowledge, this association has never been reported so far, thus it should be confirmed in a larger sample. The proximity between RA patients and healthy controls may be explained by two causes: first, the majority of our RA patients belong to a group of “early” RA with few disease symptoms which unambiguously characterize the overt RA; second, as RA is associated to a group of HLA-DRB1 alleles encoding the same epitope in the binding cleft, this subdivides the susceptibility frequency among different variants instead of just one. Moreover, the immunogenetic proximity of RA to CBD, rather than to BMD, might be the expression of the lifespan selection. This selective force has acted more on BMD than on CBD, as the formers were enrolled in adulthood, whereas the latters at birth. As a result of this selection, CBD group shows a higher frequency of disease-correlated HLA markers and a higher genetic variability compared to BMD, and this characteristic makes the choice of a CB unit more likely than adult donors in transplantation setting [26].

Finally, we revised the HLA-DQB1 typing requested for 795 celiac disease (CD) patients and 256 insulin-dependent diabetes mellitus (IDDM) patients. The frequencies of the HLA-DQB1 susceptibility alleles for CD and IDDM are listed in Table 3 for patients and controls. In CD patients the frequencies of HLA-DQB1*02:01, HLA-DQB1*02:02 susceptibility alleles were significantly higher than both control groups (*P* < 0.0001), whereas the frequency of HLA-DQB1*03:02 allele was significantly higher only versus the BMD (*P* = 0.039). The frequency of HLA-DQB1*02:01 HLA-DQB1*03:02 was significantly higher in IDDM patients than both BMD and CBD (*P* < 0.0001). 

**Figure 4:**
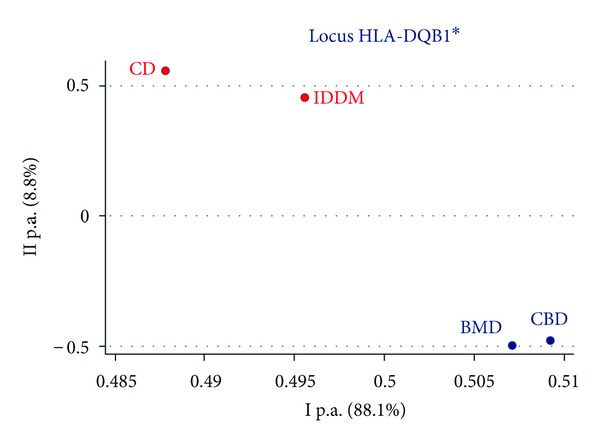
Principal component analysis of HLA-DQB1 in celiac disease (CD), insulin-dependent diabetes mellitus (IDDM), adult bone marrow donors (BMD), and cord blood donors (CBD).

HLA-DQB1 data from CD and IDDM patients were considered in the last PCoA (Figure 4). Both CD and IDDM samples are very distant from the two healthy controls, underlying a high genetic diversity between patients and controls and highlighting the appropriateness of HLA typing requests. Carriers of almost one of the HLA-DQB1 susceptibility markers were 84.91% in our CD patients (expected 95% reported in the literature) and 59.37% in IDDM (expected 60%). In both CD and IDDM, the frequency of susceptibility HLA-DQB1 alleles was in line with the literature data, and we think that the good results obtained in the analyses of HLA-DQB1-associated autoimmune pathologies may be a consequence of the correct knowledge of the marker-disease correlation for the CD and IDDM conditions.

## 4. Conclusions

Our study aims at giving a critical appraisal on the usefulness and appropriateness of the HLA typing requests in the diagnosis of HLA-associated diseases, after ten years of molecular assays typing in a single Laboratory (Immunogenetics Laboratory of the IRCCS Policlinico San Matteo of Pavia, Italy). Nevertheless, this is not an HLA-disease association study.

In this retrospective study we found that, except for HLA-DQB1 mediated pathologies (CD and IDDM), the typing requests gave an immunogenetic result less congruent than expected by the literature data. It is evident that the policy for proper typing requests needs to be improved. 

To our knowledge, this is the first survey conducted with the aim to infer the proper management of the financial resources of public health for immunogenetic testing. This is not a weird point of view if we consider two fundamental characteristics of our Hospital. First, our Immunogenetics Laboratory receives a lot of typing requests from many sources outside the Policlinico San Matteo, such as general practitioners; thus it is difficult to verify the classification criteria considered by these physicians and also their actual expertise. Secondly, our Foundation is a research hospital where patients affected by spurious syndromes or unusual cases refer to our physicians who have to deal with atypical signs and difficult diagnosis; therefore we think that the exclusion of these cases from the survey would introduce a bias in considering the total health costs for genetic testing.

In synthesis, this is an unmanipulated survey with the mere exploratory aim to improve the management of healthcare financial resources. Therefore, taking into account our data, we suggest to invest in training courses to enhance the expertise of all physicians, in particular general practitioners. To this, it is imperative to ask the Italian Society of Immunogenetics (AIBT) to set up a series of learning courses, with continuous medical education credits, to give the clinicians a precise tool to increase the appropriateness of typing requests. We believe that the interplay between clinicians and immunogenetists must be strengthened to reach a better definition of the role of HLA markers in the management of those autoimmune diseases with well-defined HLA associations.

## Figures and Tables

**Table 1:** The HLA susceptibility alleles and classification criteria references for ankylosing spondylitis, rheumatoid arthritis, uveitis, behçet's disease, celiac disease, and insulin-dependent diabetes mellitus.

Autoimmune disease	HLA susceptibility alleles	Classification criteria according to
Ankylosing spondylitis	HLA-B*27	Sieper and Rudwaleit [22]
Uveitis	HLA-B*27 HLA-DRB1*01:02, *04:05, *15:01	Deschenes et al. [23]
Behçet's disease	HLA-B*51	International Team for the Revision of the International Criteria for Behcet's Disease [24]
Rheumatoid arthritis	HLA-DRB1*01:01, *01:02, *04:01, *04:04, *04:05, *04:08, *10:01,*14:02	Silva-Fernández et al. [25]
Celiac disease	HLA-DQB1*02:01, *02:02, *03:02	Rostom et al. [26]
Insulin-dependent diabetes mellitus	HLA-DQB1*02:01, *03:02	American Diabetes Association [27]

**Table 2:** Percentage of HLA typing requests coming from medical specialists and general practitioners for each autoimmune pathology.

Autoimmune disease	*N *	HLA typing request	
		Medical specialists	General practitioners
Ankylosing spondylitis	724	45.17%	54.83%
Uveitis	125	73.60%	26.40%
Behçet's disease	212	58.49%	41.51%
Rheumatoid arthritis	554	93.86%	6.14%
Celiac disease	795	78.24%	21.76%
Insulin-dependent diabetes mellitus	256	98.44%	1.56%

**Table 3:** Allele frequencies of HLA genes correlated to autoimmune pathologies in adult bone marrow donors (BMD), cord blood donors (CBD), Behçet's disease (BD), rheumatoid arthritis (RA), celiac disease (CD), insulin-dependent diabetes mellitus (IDDM), and shared epitope (SE).

HLA susceptibility alleles	BMD	CBD	Uveitis	BD	RA	CD	IDDM
HLA-B*27	1.7%	2.15%	8.40%				
HLA-B*51	12.91%	9.88%		15.57%			
SE alleles (HLA-DRB1*01:01, *01:02, *04:01, *04:04, *04:05, *04:08, *10:01, *14:02)	10.85%	13.48%			13.72%		
HLA-DQB1*02:01	9.37%	7.92%				21.76%	17.19%
HLA-DQB1*02:02	8.99%	9.98%				17.61%	
HLA-DQB1*03:02	4.38%	5.93%				6.10%	14.45%
